# *Plasmodium simium*, a *Plasmodium vivax*-Related Malaria Parasite: Genetic Variability of Duffy Binding Protein II and the Duffy Antigen/Receptor for Chemokines

**DOI:** 10.1371/journal.pone.0131339

**Published:** 2015-06-24

**Authors:** Daniela Camargos Costa, Gabriela Maíra Pereira de Assis, Flávia Alessandra de Souza Silva, Flávia Carolina Araújo, Júlio César de Souza Junior, Zelinda Maria Braga Hirano, Flora Satiko Kano, Taís Nóbrega de Sousa, Luzia Helena Carvalho, Cristiana Ferreira Alves de Brito

**Affiliations:** 1 Laboratório de Malária, Centro de Pesquisas René Rachou, Fiocruz Minas, Belo Horizonte, Minas Gerais, Brazil; 2 FURB, Universidade Regional de Blumenau, Blumenau, Santa Catarina, Brazil; 3 CEPESBI—Centro de Pesquisas Biológicas de Indaial, Indaial, Santa Catarina, Brazil; Agency for Science, Technology and Research—Singapore Immunology Network, SINGAPORE

## Abstract

*Plasmodium simium* is a parasite from New World monkeys that is most closely related to the human malaria parasite *Plasmodium vivax*; it also naturally infects humans. The blood-stage infection of *P*. *vivax* depends on Duffy binding protein II (PvDBPII) and its cognate receptor on erythrocytes, the Duffy antigen receptor for chemokines (hDARC), but there is no information on the *P*. *simium* erythrocytic invasion pathway. The genes encoding *P*. *simium* DBP (PsDBP_II_) and simian DARC (sDARC) were sequenced from Southern brown howler monkeys (*Alouatta guariba clamitans*) naturally infected with *P*. *simium* because *P*. *simium* may also depend on the DBPII/DARC interaction. The sequences of DBP binding domains from *P*. *vivax* and *P*. *simium* were highly similar. However, the genetic variability of PsDBPII was lower than that of PvDBPII. Phylogenetic analyses demonstrated that these genes were strictly related and clustered in the same clade of the evolutionary tree. DARC from *A*. *clamitans* was also sequenced and contained three new non-synonymous substitutions. None of these substitutions were located in the N-terminal domain of DARC, which interacts directly with DBPII. The interaction between sDARC and PvDBPII was evaluated using a cytoadherence assay of COS7 cells expressing PvDBPII on their surfaces. Inhibitory binding assays *in vitro* demonstrated that antibodies from monkey sera blocked the interaction between COS-7 cells expressing PvDBPII and hDARC-positive erythrocytes. Taken together, phylogenetic analyses reinforced the hypothesis that the host switch from humans to monkeys may have occurred very recently in evolution, which sheds light on the evolutionary history of new world plasmodia. Further invasion studies would confirm whether *P*. *simium* depends on DBP/DARC to trigger internalization into red blood cells.

## Introduction

Invasion of erythrocytes by *Plasmodium vivax* merozoites is highly dependent on the interaction between the Duffy Antigen Receptor for Chemokines (DARC) and its ligand in the parasite, the Duffy binding protein (DBP) [1,2]. Individuals whose erythrocytes do not express DARC are highly resistant to invasion by *P*. *vivax* and *Plasmodium knowlesi*, a primate malaria parasite commonly found in Southeast Asia [3,4].

*Plasmodium vivax* and *P*. *knowlesi* merozoites exploit the DARC/DBP interaction to undergo internalization into several non-human primate erythrocytes, and erythrocyte susceptibility is partially dependent on the N-terminal tail of the DARC protein [2,5]. The interaction domain of DBP lies in region II of the protein, which is highly polymorphic [6–8]. This variability is associated with parasite evasion from the host immune system [9,10]. Therefore, antibodies that block DARC/DBPII interactions confer some variant-specific protection, which hampers vaccine development based on DBP [10,11]. Consequently, an understanding of erythrocyte invasion pathways of simian malaria is essential to comprehend the zoonotic potential of malaria in some regions of the world, and it might aid the development of a suitable model for the testing of drugs and vaccines against vivax malaria.

In addition to the hundreds of malaria cases that occur annually in the Amazon region (North Region of Brazil), autochthonous cases of malaria were described in the Atlantic Forest (South and Southeast of Brazil) [12–14]. Many of these cases were associated with a simian malaria parasite, *Plasmodium simium*, which is morphologically, immunologically and genetically indistinguishable from *P*. *vivax* [15–18]. This parasite was described previously as naturally infecting only two genera of monkeys, *Alouatta* (howler monkeys) and *Brachyteles* (spider monkey). Humans are susceptible to *P*. *simium*, but the natural infections described so far were accidentally acquired [14,19–23].

Our research group recently demonstrated the prevalence of *P*. *simium* infection and high levels of seropositivity against *P*. *vivax* antigens (MSP-1, DBPII and AMA-1) in wild *Alouatta guariba clamitans* from the Atlantic Forest in the Santa Catarina state in southern Brazil [24]. These findings suggested that some wild monkeys act as a *Plasmodium* reservoir for humans. *P*. *simium* infects humans, but the pathway used by this parasite to invade host erythrocytes has not been identified. We investigated the genetic variability of binding domains from *P*. *simium* DBPII (PsDBPII) and simian DARC on erythrocytes from *Alouatta g*. *clamitans* (sDARC) to gain insight into the evolution of *Plasmodium* species in the New World. We also aimed to gain insight into *P*. *simium* DBP and simian DARC interactions.

## Materials and Methods

### Animal information

Captive and wild *Alouatta g*. *clamitans* (Southern brown howler monkeys) were studied. Captive monkeys were obtained from the Conservationist Center of Biological Research at Indaial—CEPESBI (IBAMA Registration No. 1/42/98/000708-90) in the Indaial municipally, and wild monkeys were obtained from the Geisler Mountain in the surroundings areas of Indaial in the Santa Catarina state in southern Brazil. CEPESBI is a scientific captivity center of the University of Blumenau for the maintenance and conservation of *A*. *clamitans*. It is part of the Southern Brown Howler Project, which also has an observatory of non-human primates at Geisler Mountain connected to the National Park of Serra do Itajaí, part of the Atlantic Forest. CEPESBI researchers follow approximately 60 red howler monkeys, and 20 monkeys were caught for sample collection. Free-living animals were captured using anesthetic darts (tiletamine and zolazepam 3.9 mg/kg) with the aid of a compressed air gun (Dist-Inject moldel 70). The captivity area has 45 red howler monkeys that were obtained from different places around the municipality of Indaial, and these monkeys have been maintained in captivity from one year to more than 10 years. Animals were housed in couples in 3 m x 5 m x 2.6 m rooms containing ropes, logs and feeding platforms, and they were fed six times daily with a diet consisting of a variety of fruits and vegetables, a commercial ration (Nuvilab CR- 1, Sogorb, São Paulo, Brazil), leaves of *Cecropia* sp. and *Sechium edule* and water *ad libitum*. No animals were sacrificed in this study, and all free-ranging animals returned to their natural habitat after sample collection. The Brazilian government authorized this study and the access to and transport of biological samples through SISBIO no. 28953-1/2011 and 28953-2/2012. The Ethics Committee of the Regional University of Blumenau approved this study (no.003/12 and no. 516/2012).

### Sample collection, diagnosis and DNA extraction

Captive animals were contained using dip nets and sedated via intramuscular administration of a combination of tiletamine and zolazepam. A veterinarian from CEPESBI obtained blood samples (5 mL) with and without anticoagulant (EDTA) using femoral venipuncture from each animal. The heart rate, breathing, eye reflexes and temperature were monitored during sedation. Only one animal showed symptoms related to malaria as previously described by us [24]. The other animals were healthy at the time of blood collection. DNA extraction from blood samples was performed using the Gentra Puregene Blood kit (Qiagen, Venlo, Netherlands) according to the manufacturer’s recommendations. Infection was identified in 9 animals (2 captive and 7 wild) using microscopy of panoptic-stained thin blood smear and confirmed using molecular techniques including nested PCR and real-time PCR as *P*. *vivax*/*P*. *simium* [24].

### *Plasmodium simium dbp*II amplification

Extracted DNA samples from infected monkeys were used as a template in PCR to amplify a fragment of the *P*. *simium* DBPII-encoding gene as previously described [8]. Platinum high fidelity Taq DNA polymerase (1 U) (Invitrogen Life Technologies, Thermo Fisher Technologies, Grand Island, NY, USA) was used in a 10 μL PCR reaction with 100–200 ng DNA, 0.125 mM dNTPs, 0.5 μM primers (forward and reverse for *P*. *vivax dbpII*), 0.75 mM MgCl_2_, and 1 μL enzyme buffer. The following amplification parameters were used: 94°C for 3 min, 35 cycles of 94°C for 30 sec, 61°C for 30 sec and 72°C for 2 min, followed by a last extension cycle of 72°C for 5 min.

### DARC amplification

Two pairs of primers were used to amplify two fragments of the gene encoding DARC from *A*. *g*. *clamitans* (sDARC1 and sDARC2). sDARC1 corresponding to the 5’ end of the gene, which includes the promoter, exon 1 (codons 1 to 7) and part of the intron (positions -154 to 157 of *Homo sapiens* DARC—hDARC, accession number JN251915.1), was amplified using primers KAT031 and KAT035 described by Demogines et al. [5]. The 20 μL reaction contained 100–200 ng of DNA, each primer at 0.5 μM, 0.125 mM dNTPs, 2 mM MgCl_2_, 1 U Taq DNA polymerase and 2 μL of enzyme buffer. PCR cycling was 94°C for 3 min, 35 cycles of 94°C for 40 sec, 64°C for 40 sec and 72°C for 1 min, followed by a final cycle of 72°C for 5 min. DARC2 includes part of exon 2 (nucleotides 1254 to 2072 of *Saimiri sciureus* DARC, accession number HQ285857.1), and it was amplified using primers DARC2F 5’CCCCTCCCACCTGCCCC3’ and DARC2R 5’GCCACCAGAAAATAAACCAG3’ in the same conditions described above, except for the annealing temperature of 60°C.

### Sequencing of PCR-amplified DNA

PCR products were purified using the Purification Kit (Qiagen Inc., Valencia, CA, USA) following the manufacturer’s procedure. Approximately 1–10 ng of purified PCR products was amplified using each primer at 3.3 μM (forward or reverse) and 1 μL of Big Dye terminator kit (Life Technologies) in a program of 96°C for 1 min, 35 cycles of 96°C for 15 sec, the temperature of primer annealing for 15 sec and 60°C for 15 sec. The fragments were precipitated using ammonium acetate, resuspended in formamide HI-DI (Applied Biosystems, Thermo Fisher Scientific, Inc., Waltham, MA, USA) and analyzed in an ABI 3730 DNA (Applied Biosystems) automatic sequencer.

### Serological assay

An ELISA (enzyme-linked immunosorbent assay) to detect IgG antibodies in monkey sera was performed using recombinant *P*. *vivax* Duffy binding protein (rDBP—regions II to IV) as previously described [24]. rDBP was used at a final concentration of 5 μg/mL with serum samples assayed at a 1:100 dilution, and *Macaca mulatta* anti-IgG was used as the secondary antibody (Sigma-Aldrich, St. Louis, MO). The cut-off was based on the mean plus three standard deviations of sera reactivity from 8 non-exposed monkeys, and the results are expressed as a reactivity index (IR = OD_492nm_ values of test sample divided by the value of the cut-off).

### COS-7 cell transfection and erythrocyte-binding assays

Sal-1 PvDBP-pEGFP recombinant plasmid [25,26] was transfected into COS-7 cells (American Type Culture Collection, Manassas, VA, USA) using lipofectamine and PLUS-reagent (Invitrogen Life Technologies, Carlsbad, CA, USA) according to the manufacturers’ protocols. Briefly, COS-7 cells in six-well culture plates (1.5 × 10^5^ cells/well) were transfected with plasmid (0.5 μg/well)-liposome (5% Plus-reagent and 3% lipofectamine) complexes in Dulbecco’s Modified-Eagle Medium (DMEM, Sigma, St. Louis, MO, USA) without serum. Transfection medium was replaced by DMEM with 10% of fetal bovine serum (Gibco-BRL, Gaithersburg, MD, USA) after 6 h of cell exposure to the DNA liposome complexes (37°C, 5% CO_2_). Culture medium was replaced again 24 h after transfection, and the efficiency of transfection was assessed using fluorescence. Erythrocyte-binding assays were performed 48 h after transfection as previously described [27]. Briefly, erythrocytes from two monkeys (BL10 and BL34) were added to the cell cultures, and plates were incubated for 2 h at room temperature. Unbound erythrocytes were removed by washing the wells three times with phosphate-buffered saline (PBS). Binding was quantified by counting the number of rosettes observed over 10–20 fields of view (×200). Positive rosettes were defined as adherent erythrocytes that covered more than 50% of the COS cell surface.

### Inhibition of erythrocyte binding assays

Blockade of erythrocyte binding was performed using sera of infected and non-infected monkeys added at 1:30, and the plates were incubated for 1 h at 37°C in 5% CO_2_. The 1:30 dilution was chosen because it provided a wide range of inhibitory activity in previous experiments (data not shown). Human O^+^ erythrocytes from a DARC-positive individual in a 10% suspension were added to each well (200 μL/well), and the plates were incubated for 2 h at room temperature. Unbound erythrocytes were removed by washing the wells three times with PBS. Binding was quantified by counting the number of rosettes observed over 10–20 fields of view (×200). Positive rosettes were defined as adherent erythrocytes that covered more than 50% of the COS cell surface. DARC-negative human serum was used as a negative control for each assay. The percent inhibition was calculated as 100 × (*R*_*c*_—*R*_*t*_)/*R*_*c*_, where *R*_*c*_ is the average number of rosettes in the control wells, and *R*_*t*_ is the average number of rosettes in the test wells.

### Analyses

Identity of DNA sequences was confirmed using the BLAST program (www.ncbi.nlm.nih.gov/BLAST). DNA sequences were analyzed using BioEdit sequence alignment editor (www.mbio.ncsu.edu/BioEdit/bioedit.html) to align the sequences and identify polymorphisms. Genetic diversity was analyzed using DnaSP version 5.10 [28]. The three-D structure of the PvDBPII dimer (PDB– 3RRC) [29] was visualized using PyMol v1.1 [30], and it was used to identify polymorphic residues. Phylogenetic analyses were performed using the Maximum likelihood method with Tamura—Nei model and 5,000 bootstrap replicates in MEGA 5.2 [31]. Statistical analyses were performed using STATA software v12.

## Results

### Single polymorphic nucleotides in Duffy binding protein II of *Plasmodium simium* (PsDBPII)

**Fig 1:**
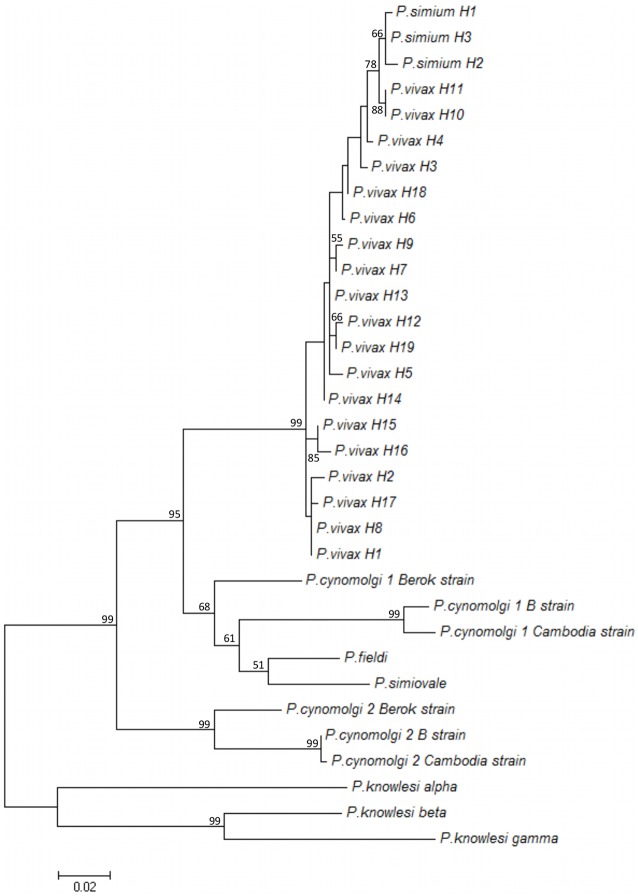
Phylogenetic tree of *dbpII* from the 19 most predominant *P*. *vivax* haplotypes. Haplotypes with > 1% frequency were used here: accession numbers (haplotype). *P*. *vivax dbp*: EU812840.1 (H1), EU812841.1 (H2), EU812842.1 (H3), EU812844.1 (H4), EU812845.1 (H5), EU812849.1 (H6), EU812861.1 (H7), EU812869.1 (H8), EU812874.1 (H9), EU812898.1 (H10), EU812915.1 (H11), EU812927.1 (H12), EU812954.1 (H13), AF220662 (H14), AF289650 (H15), AF289649 (H16), GU143965 (H17), GU143986 (H18), EF379128 (H19); *P*. *cynomolgi dbp*1: AB617788.1 (Cambodia strain), JQ422035.1 (Berok strain), XM_004221494.1 (B strain); *P*. *cynomolgi dbp2*: AB617789.1 (Cambodia strain), JQ422036.1 (Berok strain), XM004220981.1 (B strain); *P*. *knowlesi dbp*: M90466.1 (alpha), M90694.1 (beta) and (M90695.1 gamma); *P*. *fieldi dbp*: AB617790.1; *P*. *simiovale dbp*: AB617791.1; and three haplotypes of *P*. *simium* isolates herein (H1, H2, H3). The tree was build using Maximum likelihood method with the Tamura and Nei model (TN93 model) in Mega 5.2. Numbers in the nodes indicate bootstrap values greater than 50.

**Fig 2:**
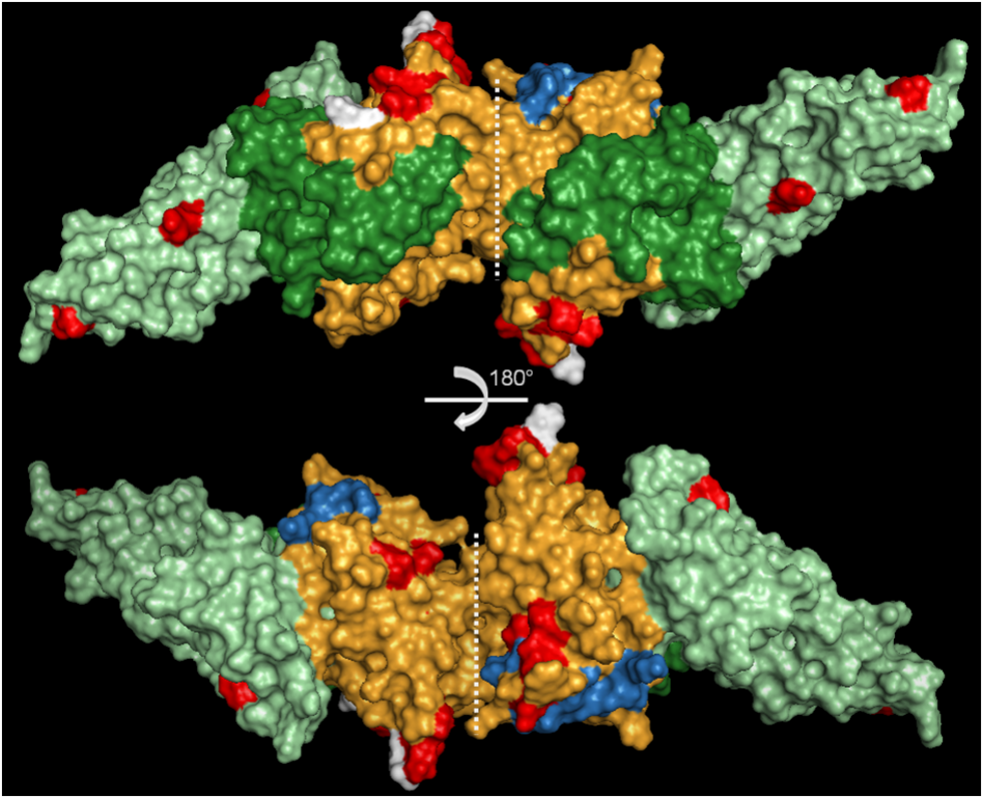
Three-dimensional structure of the PvDBPII dimer. PvDBPII dimer (3RRC) showing the polymorphic sites in *P*. *vivax* (red) and *P*. *simium* (white). The three subdomains of the protein are shown in green (subdomain 1), orange (subdomain 2) and light green (subdomain 3). Residues important for the DARC interaction are shown in blue. 3-D structure visualized using PyMol. The structures correspond to a 180° rotation in the horizontal plan.

*Plasmodium simium* infection was detected in 9 of 65 monkeys (2 of 45 captive and 7 of 20 wild monkeys) using molecular diagnosis [24]. The DNA samples were further used to amplify PsDBPII. Good quality sequences of the PsDBPII-encoding gene (positions 1010–1447 of *P*. *vivax* Sal-1, accession number M61095.1) were obtained in 7 of 9 infected monkeys. Four polymorphic sites were identified in the *P*. *simium* sequences (nucleotide positions 1016, 1113, 1153 and 1154 of PvDBP sequence), and these sites all contained non-synonymous substitutions (S1 Fig). These polymorphisms were combined in three distinct haplotypes of PvDBPII (Table 1). Comparisons to the most prevalent *P*. *vivax dbpII* sequences available in GenBank identified 22 SNPs in *P*. *simium dbp* sequences (S1 Fig). The substitution 1233 A > C was present in all *P*. *simium* isolates, and it was never identified in *P*. *vivax* isolates worldwide. Comparisons of the genetic diversity of *dbpII* sequences from *P*. *simium* and all *P*. *vivax* sequences available in GenBank identified 84 polymorphic sites with a nucleotide diversity (π) of 0.0124 (Table 2). The comparison of *P*. *simium* sequences with a *P*. *cynomolgi dbpII* sequence allowed the identification of 56 polymorphic sites (π = 0.0348), and comparison with a *P*. *knowlesi dbp* alpha sequence identified 86 polymorphic sites (π = 0.0526). The nucleotide diversity of *dbp* sequences from *P*. *vivax*/*P*. *simium* (π = 0.0124) was slightly lower than those of *P*. *vivax*/*P*. *cynomolgi* (π = 0.0127) and *P*. *vivax*/*P*. *knowlesi* (π = 0.0130) (Table 2). Haplotype diversity was quite high for all species, but this result must be analyzed with caution because of the high dependency of the experimental sample size. Phylogenetic analyses were performed to identify relationships between *dbpII* sequences from different *Plasmodium* species. The three *dbpII* haplotypes from *P*. *simium* clustered together, and this clade exhibited a close relationship with *dbpII* from *P*. *vivax* predominant haplotypes (Fig 1). Polymorphic sites of *P*. *simium* and *P*. *vivax* were mapped on the PvDBPII 3D-structure (Fig 2). Polymorphisms of *P*. *simium* and *P*. *vivax* were concentrated in DBPII sub-domain 2. However, *P*. *simium* SNPs were not located near the DARC binding site, unlike the most important *P*. *vivax* polymorphisms.

**Table 1:** Single nucleotide polymorphisms (SNPs) in the ligand domain of the Duffy binding protein (DBPII)-encoding gene in seven isolates of *Plasmodium simium*.

		Nucleotide* (AA)		
Monkey code	Haplotype	1016 (338)	1113 (371)	1153–1154 (385)
BL3, BL10	1	AGA (R)	AAA (K)	ATA (I)
BL4, BL5, BL61, BL64	2	AAA (K)	AAT (N)	CAA (Q)
BL6	3	AGA (R)	AAA (K)	AAA (K)

* Positions corresponding to *P*. *vivax* Sal-1 *dbp*, accession number M61095.1.

Underlined residues are polymorphic. Dots indicate the same residue in the sequences compared to the reference sequence.

**Table 2:** Genetic diversity of *P*. *simium* DBPII compared to other *Plasmodium* DBPII.

Group	*Plasmodium* species (N)	S	Singleton variable sites	Parsimony informative sites	π ± SD	Synonymous changes	Non-synonymous changes	H	Hd ± SD
1	*P*. *simium* (7)	3	0	3	0.0038 ± 0.0008	0	3	3	0.6670 ± 0.1600
2	*P*. *simium* + *P*. *vivax* ^*a*^ (8)	10	7	3	0.0080 ± 0.0031	1	7	4	0.7500 ± 0.1390
3	*P*. *simium* + *P*. *cynomolgi* ^*b*^ (8)	56	53	3	0.0348 ± 0.0224	17	39	4	0.7500 ± 0.1390
4	*P*. *simium* + *P*. *knowlesi* ^*c*^ (8)	86	83	3	0.0526 ± 0.0352	32	52	4	0.7500 ± 0.1390
5	*P*. *vivax* (511)	81	42	39	0.0122 ± 0.0003	14	64	118	0.9196 ± 0.0078
6	*P*. *vivax* + *P*. *simium* (518)	84	42	42	0.0124 ± 0.0003	14	65	121	0.9217 ± 0.0077
7	*P*. *vivax* + *P*. *cynomolgi* ^*b*^ (512)	122	81	41	0.0127 ± 0.0005	20	87	119	0.9199 ± 0.0078
8	*P*. *vivax* + *P*. *knowlesi* ^*c*^ (512)	145	101	44	0.0130 ± 0.0008	34	91	119	0.9199 ± 0.0078

^a^ *P*. *vivax* Sal-1, accession number M61095.1.

^b^ *P*. *cynomolgi* 1, accession number XM004221494.1.

^c^ *P*. *knowlesi* alpha, accession number M90466.1.

N: number of sequences; S: segregating sites; π: nucleotide diversity; H: haplotypes; Hd: haplotype diversity; SD: standard deviation

### Characterization of sDARC

**Fig 3:**
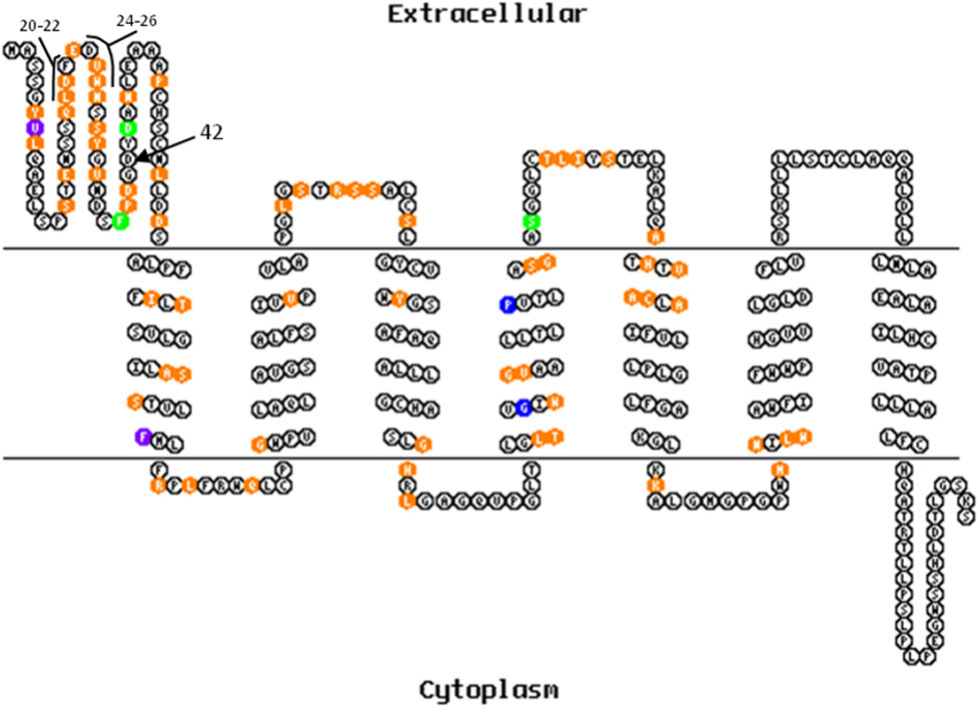
Schematic model of DARC showing polymorphic sites. The 2-D model of DARC from *Homo sapiens* was constructed using TOPO2 software. Polymorphic sites in primates are shown in orange, purple (SNPs exclusive to the Cercopithecidae family), green (SNPs exclusive to the Platyrrhini parvorder) and blue (SNPs exclusive to the Hominoidea superfamily). Residues involved in direct binding to DARC are indicated (20–22 and 24–26) according to Batchelor et al. [53]. The arrow indicates Asp42Gly. Polymorphisms were annotated only until codon 255 (fragment available in our sequences).

Fragments of the sDARC-encoding genes (promoter and nucleotides 1 to 768) from 10 *A*. *g*. *clamitans* individuals were sequenced, and no polymorphisms were identified (data not shown). Comparisons to the other primate DARC sequences available in GenBank identified 222 polymorphic sites (S2 Fig). *Alouatta g*. *clamitans* exhibited 7 SNPs that were not previously identified in any other primate species, and 3 of these SNPs were non-synonymous substitutions (at nucleotide positions 530, 745 and 746) (S2 Fig). N-terminal sDARC (aa 1–42) exhibited the highest genetic variability (Table 3). Polymorphic sites were not only concentrated in this region but were distributed throughout the entire molecule, including the transmembrane segments (Fig 3). The mutation indicative of a null allele (-67 T > C) (data not shown) and the substitution responsible for the FY*A allele (Asp42Gly) were not observed in our *A*. *clamitans* DARC sequences (S2 Fig).

**Table 3:** Genetic diversity of DARC from Southern brown howler monkeys (*Alouatta g*. *clamitans)*.

DARC (AA)	S	Singleton variable sites	Parsimony informative sites	π ± SD	Synonymous changes	Non-synonymous changes
1–42	54	25	29	0.07499 ± 0.00642	17	14
43–256	168	75	93	0.04622 ± 0.00268	88	64
1–256	222	100	122	0.05088 ± 0.00265	105	78

AA: amino acid position; S: number of segregating sites; π: nucleotide diversity; H: haplotypes; Hd: haplotype diversity; SD: standard deviation.

**Fig 4:**
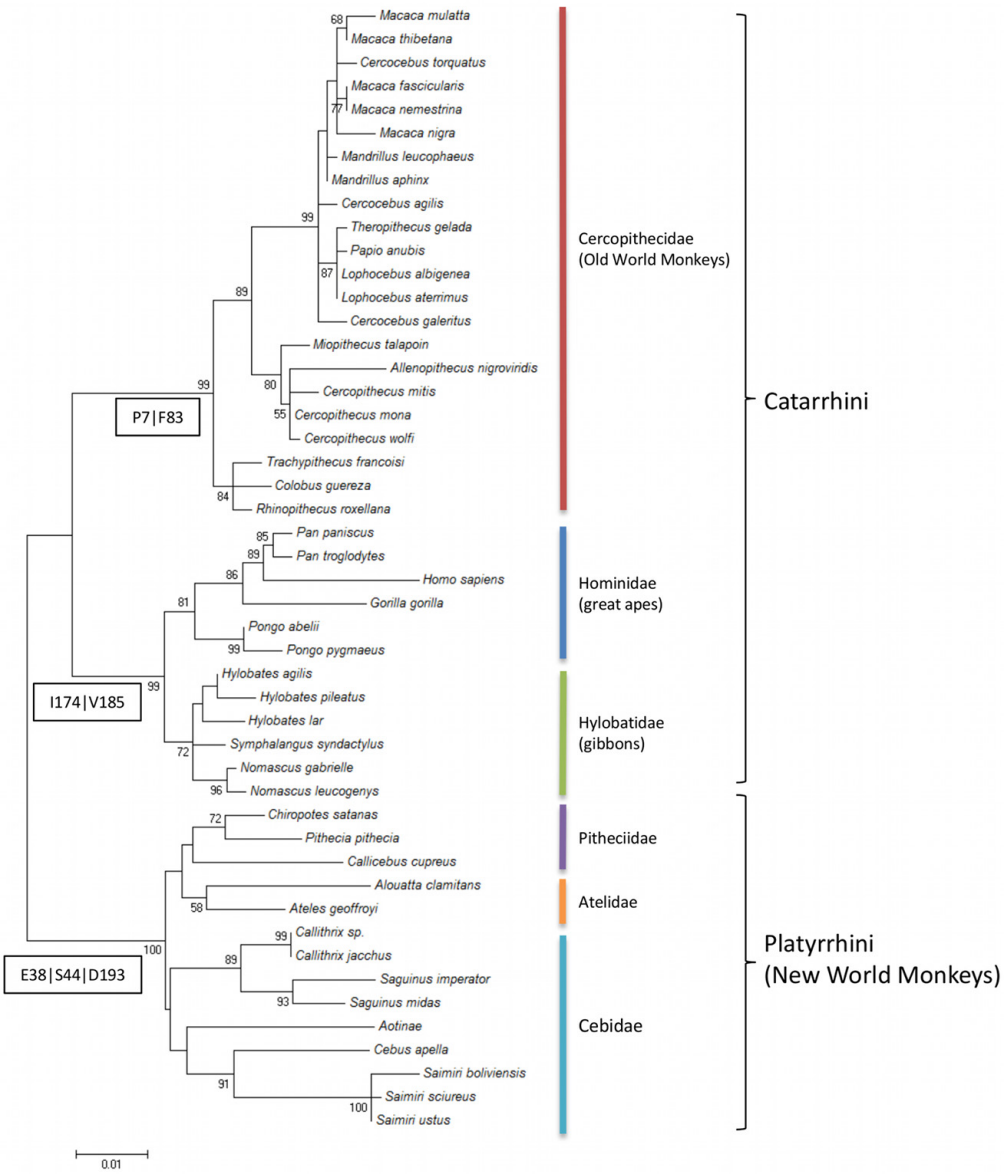
Phylogenetic tree of DARC from primates. Sequences of DARC from 47 primate species available from GenBank were aligned with the *Alouatta g*. *clamitans* DARC sequences described here. Clusters of parvorder: Platyrrhini and Catarrhini, and Families: Cercopithecidae, Hominidae, Hylobatidae, Pitheciidae, Atelidae and Cebidae are indicated. The numbers in the boxes correspond to the exclusive codons from each group (one letter amino acid and position).

The reconstruction of the phylogenetic relationships of primates based on the N-terminal DARC sequence (responsible for DBP binding) clustered *A*. *g*. *clamitans* with *Ateles geoffroyi* in the clade of Platyrrhini parvorder (New World monkeys) (Fig 4). Notably, only a few SNPs were exclusive to each group: two from the Cercopithecidae family (P7 and F83), two from the Hominoidea superfamily (I174 and V185) and three from the Platyrrhini family (E38, S44 and D193) (Figs 3 and 4).

### DARC/DBP *in vitro* interaction and inhibitory antibodies

**Fig 5:**
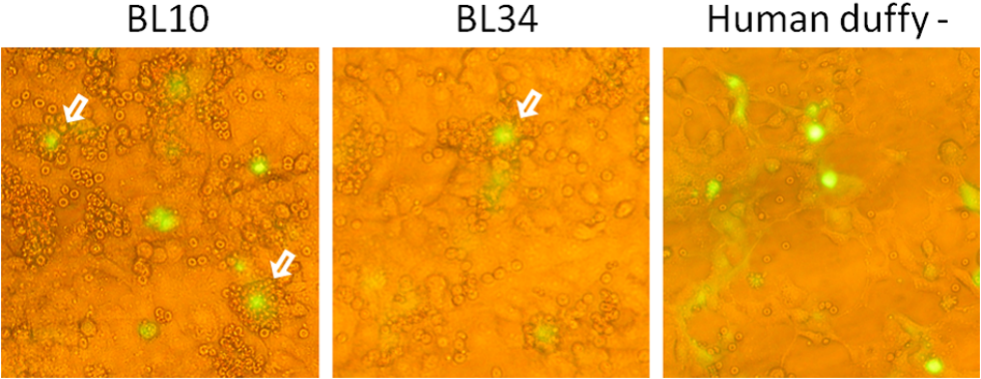
Interaction of *Alouatta g*. *clamitans* DARC and PvDBPII. (A) Interaction between *P*. *vivax* DBPII expressed on the COS7 cell surface and DARC on the surface of *A*. *clamitans* erythrocytes (BL10 and BL34), representative of two experiments. Green fluorescence indicates transfected cells expressing GFP. (B) The negative control was performed using human DARC-negative erythrocytes. The cells were observed using conventional epifluorescence microscopy (×200). White arrows indicate the rosettes.

We next investigated whether monkey sera blocked the interaction between human DARC (hDARC) and PvDBPII. A preliminary experiment investigated whether sDARC from *A*. *clamitans* bound DBPII from *P*. *vivax*. The results clearly demonstrated that simian erythrocytes (all DARC positive) recognized PvDBPII on the surface of COS-7 cells. Fig 5A illustrates one of two experiments in which monkey erythrocytes formed rosettes in COS-7 cells expressing PvDBPII. hDARC-negative erythrocytes exhibited no binding to DBPII expressed on COS-7 cells (Fig 5B).

We performed *in vitro* erythrocyte binding inhibition assays, in which sera of infected-monkeys were used to block the interaction between hDARC/PvDBPII, to further investigate the inhibitory activity of immune plasma samples. We selected 8 monkeys (acute or chronically infected) with different levels of anti-PvDBP ELISA-detected IgG antibodies (Table 4). Binding inhibition ranged from 0 to 94%, with one monkey (BL37) showing the strongest DARC/DBP inhibition. Titration of the inhibitory antibody responses of the two highest inhibitory sera exhibited a dose-response curve (S3 Fig). A positive correlation was identified between the reactivity index in ELISA and the percentage of DARC/DBP interaction blockade (Spearman’s correlation coefficient = 0.843, P = 0.011).

**Table 4:** Characteristics of immune sera from monkeys used for the inhibition of the hDARC/PvDBPII interaction.

Monkey Code	Acute infection^b^	Anti-PvDBPII IgG, RI^c^	Percentage of inhibition
**BL69**	Pos	22	44%
**BL64**	Pos	18	40%
**BL10** ^**a**^	Pos	12	36%
**BL34**	Neg	22	43%
**BL37**	Neg	14	94%
**BL40**	Neg	7	10%
**BL41**	Neg	6	0
**BL44**	Neg	1	0

^a^BL10 was the only symptomatic animal [24]

^b^Peripheral blood infection was detected using conventional microscopy, and *P*. *vivax/P*. *simium* identification was confirmed using amplification of the *P*. *vivax* 18SSU rRNA gene with two different PCR-based protocols (Nested and Real-time) [24]

^c^Anti-PvDBPII IgG antibodies were detected by ELISA using recombinant PvDBP (aa 132–771)[24], and the results are expressed as Reactivity Index (RI), which corresponds to OD_492nm_ values of test samples divided by the cut-off value.

Values of RI > 1 were considered positive.

## Discussion

The major pathway for erythrocyte invasion by *P*. *vivax* merozoites is dependent on the interaction between parasite DBPII and DARC on the host cell surface [32]. This dependency and the capacity of DBP to elicit a protective immune response rendered DBP one of the most prominent vaccine candidates against malaria caused by *P*. *vivax* [33–36]. The elicited immunity is related to the genetic diversity of the DBP ligand domain, and blockade of the DBPII/DARC interaction appears to be variant-specific. The close proximity between *P*. *vivax* and *P*. *simium* suggests that the DBPII/DARC pathway is also involved in *P*. *simium* erythrocyte invasion. We analyzed the DBPII sequences from isolates of *P*. *simium* from seven naturally infected monkeys to gain insight into the genetic variability of *P*. *simium* DBPII. The results demonstrated much less variability in PvDBPII than its orthologous protein in *P*. *vivax* available in GenBank. Numerous segregating sites and a high overall nucleotide diversity in *P*. *vivax* DBPII [37] were not observed for *P*. *simium* DBPII. This result corroborates the hypothesis of a recent transfer of *Plasmodium* species from humans to New World monkeys (Atelidae family), as previously suggested [38–44]. Tazi and Ayala [43] and Lim et al. [44] noted biological and historical reasons in favor of the transfer from humans to New World monkeys, such as the worldwide distribution of *P*. *vivax* compared to a very limited geographic description of *P*. *simium* and the restricted number of natural hosts of *P*. *simium*. We cannot disregard the possibility that this low variability does not mean that *P*. *simium* is evolutionarily more recent than *P*. *vivax*, but it could be a consequence of a recent bottleneck in the *P*. *simium* population, which is consistent with the absence of synonymous substitutions observed in PsDBPII. However, the high genetic identity found here between *P*. *simium* and *P*. *vivax* DBPII reinforces their close phylogenetic relationship and strengthens the hypothesis that the host switch arose in very recent evolutionary times. This switch may have occurred only after the latest five centuries of European colonization [44,45]. Accordingly, the genetic identity previously reported for *P*. *vivax* and *P*. *simium* at rapidly evolving loci, such as microsatellites and tandem repeats, confirms that the host transfer must have occurred recently in the evolutionary scale [40].

Similar to *P*. *vivax*, *P*. *simium* exhibited an excessive number of non-synonymous substitutions in DBPII, which suggests diversification due to selective pressure by the host immune response [37,46]. The spatial localization of polymorphic residues of *P*. *vivax* DBPII near the erythrocyte-binding domain, in which substitutions divert recognition by inhibitory antibodies, likely contributes to the variant specificity of the immune response [8,47]. Notably, polymorphic residues of PsDBPII were distinctly localized from the *P*. *vivax* polymorphic residues in the DBPII 3-D structure. Despite this localization difference, we identified at least one *A*. *clamitans* antibody in sera that strongly inhibited (94%) the *in vitro* interaction between PvDBPII and human DARC.

The *P*. *simium* erythrocyte invasion pathway was not the focus of the current work, but the availability of transfected COS cells expressing PvDBPII [48] offered an opportunity to gain insight into the biology of invasion. The *in vitro* experiments demonstrated that *A*. *clamitans* erythrocytes bound to PvDBPII, and this *in vitro* interaction, as evaluated by the number of rosettes, was similar to that with human erythrocytes (data not shown). Most interestingly, the results demonstrated that simian immune sera inhibited the interaction between hDARC and PvDBPII. ELISA reactivity positively correlated with the inhibition levels, but great variability in the profile of inhibitory response was observed, and most of the monkeys generated weak or non-neutralizing antibodies to DBPII. We cannot exclude the possibility that the use of a homologous system (PsDBP/sDARC) could increase this response. However, these results are consistent with *P*. *vivax*-exposed populations, which showed that PvDBPII was weakly immunogenic and induced strain-specific immunity [10,49]. Consequently, it was not surprising that a single animal produced high levels of binding inhibitory antibodies (BIAbs) that cross-reacted with PvDBPII. These findings are interesting because few infected people developed a significant BIAbs response (defined as “elite responders”) and produced broadly reactive anti-DBPII binding inhibitory antibodies that were directed against more conserved B cell epitopes ([48], revised in [50]). Therefore, these results reinforce the possibility of using *P*. *simium/*New World monkeys as a promising tool to guide the development of DBPII as a vaccine against *P*. *vivax*.

Although more definitive invasion/inhibitory assays are needed to confirm whether *P*. *simium* infection depends on the DBP/DARC interaction, this invasion pathway is already well-established in another simian malaria parasite, *P*. *knowlesi* [51], which is also able to infect humans [52] but is evolutionarily less closely related to *P*. *vivax* than *P*. *simium* [53]. This parasite has two paralogous erythrocyte binding proteins, EBP β and γ, in addition to the orthologous DBP PkEBP α. These two *P*. *knowlesi* EBP proteins, which do not bind DARC positive-erythrocytes, exhibited much higher diversity than PvDBP than PsDBP [37], which corroborates the potential to use *P*. *simium* as a model for *P*. *vivax* infection.

Phylogenetic analysis of the sDARC fragment (aa 1 to 255) showed very good correspondence with highly resolved primate phylogeny [54]. DARC exhibited a high level of variability among the 48 primates, and the N-terminal domain (aa 8 to 42), which was identified as the binding domain for *P*. *vivax* erythrocyte invasion, was the most polymorphic protein region [55]. This finding reinforces the hypothesis of selective pressure acting in this domain, as previously demonstrated [5,56]. Recently, the structural analysis of PvDBP/hDARC binding defined amino acids 19 to 30 of DARC as a critical interaction site, in which L20/D21/F22 and D24/V25/W26 residues make direct contact with DBPII [57]. Different combinations of amino acids in these positions according to the group of primates are found (S2 Fig), and the most polymorphic residue was at position 25. This residue was implicated as an inter-species barrier to *P*. *vivax* invasion because *Gorilla gorilla* has mutation V25A, which disrupts an essential hydrophobic interaction with PvDBP that stabilizes DARC/DBP binding [5,57,58]. All New World monkeys exhibited a V25L mutation, which is expected to maintain the stabilization role of this residue, which suggests that all of these monkeys are susceptible to *P*. *vivax* invasion. This result indicates that there is not a clear signature in DARC for the high invasion dependency observed in some *Plasmodium* species, such as *P*. *vivax* and likely *P*. *simium*. Therefore, this feature does not have a monophyletic origin.

Beyond the evolutionary importance of simian malaria, the existence of a sylvatic *P*. *simium* reservoir of malaria infection might have public health implications. The presence of *P*. *vivax-*related parasites in monkeys was also described in Central Africa, where human *P*. *vivax* infection is not common because of the high levels of Duffy negativity in the human population [59]. This result suggests that humans living in close proximity to monkeys in specific geographic areas, such as the Atlantic Forest in Brazil and Central Africa, may harbor *P*. *vivax*-related simian malaria. Antibodies against *P*. *vivax* pre-erythrocytic stages were described in DARC- negative individuals in the Republic of Congo (West Central Africa), and *P*. *vivax* autochthonous cases may be common in travelers returning from this region [60]. Autochthon cases of malaria may have also originated in the Atlantic Forest area in Brazil [11–13]. Therefore, the potential of simian *Plasmodium* infections as a malaria reservoir should be considered in the international effort to reduce the global incidence of malaria.

## Conclusions

The close phylogenetic relationship between *P*. *simium* and *P*. *vivax* and the lower DBPII variability in infected monkeys suggest a recent host switch from New World monkeys to humans. The possibility of a sylvatic reservoir of Plasmodium species in Atlantic Forests should be considered in the current efforts to control and eliminate human malaria. Furthermore, the possibility that *P*. *vivax* and *P*. *simium* share a similar erythrocyte invasion pathway, which was reinforced by the presence of inhibitory antibodies in wild *A*. *clamitans*, makes the *P*. *simium*/New World monkeys an attractive model for vaccine and drug development. Towards that goal, it will be important to validate this model using *in vitro* and *in vivo* assays, particularly using small New World monkeys, such as capuchin monkeys (subfamily Cebinae), which we found to be naturally infected with *P*. *simium* [61].

## Supporting Information

S1 Fig Multiple alignment of *dbpII* partial sequences from *P*. *vivax* and *P*. *simium*. *dbpII* sequences (positions 1021 to 1300 of *P*. *vivax* Sal-1 *dbp*, accession number M61095.1) from the 19 most prevalent haplotypes of *P*. *vivax* (sequences with > 1% frequency) in 511 available sequences, accession numbers (haplotype): EU812840.1 (H1), EU812841.1 (H2), EU812842.1 (H3), EU812844.1 (H4), EU812845.1 (H5), EU812849.1 (H6), EU812861.1 (H7), EU812869.1 (H8), EU812874.1 (H9), EU812898.1 (H10), EU812915.1 (H11), EU812927.1 (H12), EU812954.1 (H13), AF220662 (H14), AF289650 (H15), AF289649 (H16), GU143965 (H17), GU143986 (H18), EF379128 (H19); and the seven *P*. *simium dbp* sequences (herein). White head arrows indicate polymorphic sites, and black head arrows indicate polymorphisms among *P*. *simium* sequences. Identical residues are represented by dots. An asterisk shows the species-specific polymorphism (1233A>C).
(PDF)

S2 Fig Multiple alignments of *darc* partial sequences. *darc* sequences of *Alouatta g*. *clamitans* were aligned with other primate *darc* sequences available in GenBank (nucleotides 1 to 768, from *Homo sapiens darc* sequence, accession number JN251915.1). The numbered bars above the sequences indicate transmembrane domains. The grey box shows the N-terminal minimum-binding domain (19–30 aa), and residues responsible for direct interaction with DBPII are underlined (20,21,22 and 24,25,26). Colored bars on the left side of the alignment represent the phylogenetic group families: red (Cercopithecidae), blue (Hominidae), green (Hylobatidae), purple (Pitheciidae), orange (Atelidae) and aqua (Cebidae). The arrow indicates the polymorphism Asp42Gly, which is responsible for the FY*A and FY*B alleles.
(PDF)

S3 Fig Titration of the inhibitory serum responses. The sera from two monkeys (BL37 and BL69), which showed the highest levels of blockade, were used in dilutions of 1:30, 1:90 and 1:270 in the inhibition assays of human DARC and PvDBPII. Inhibition was calculated based on the reduction in the rosette numbers observed in the presence of monkey serum compared to the rosette numbers in the absence of serum.
(TIF)
