# Supplementary material for: Plasmodium simium, a Plasmodium vivax-Related Malaria Parasite: Genetic Variability of Duffy Binding Protein II and the Duffy Antigen/Receptor for Chemokines
Source: PLoS One. 2015 Jun 24;10(6):e0131339. doi: 10.1371/journal.pone.0131339 (PMC4480967; doi:10.1371/journal.pone.0131339)
Supplement: S1 Fig — dbpII sequences (positions 1021 to 1300 of P. vivax Sal-1 dbp, accession number M61095.1) from the 19 most prevalent haplotypes of P. vivax (sequences with > 1% frequency) in 511 available sequences, accession numbers (haplotype): EU812840.1 (H1), EU812841.1 (H2), EU812842.1 (H3), EU812844.1 (H4), EU812845.1 (H5), EU812849.1 (H6), EU812861.1 (H7), EU812869.1 (H8), EU812874.1 (H9), EU812898.1 (H10), EU812915.1 (H11), EU812927.1 (H12), EU812954.1 (H13), AF220662 (H14), AF289650 (H15), AF289649 (H16), GU143965 (H17), GU143986 (H18), EF379128 (H19); and the seven P. simium dbp sequences (herein). White head arrows indicate polymorphic sites, and black head arrows indicate polymorphisms among P. simium sequences. Identical residues are represented by dots. An asterisk shows the species-specific polymorphism (1233A>C). (PDF) [file pone.0131339.s001.pdf]

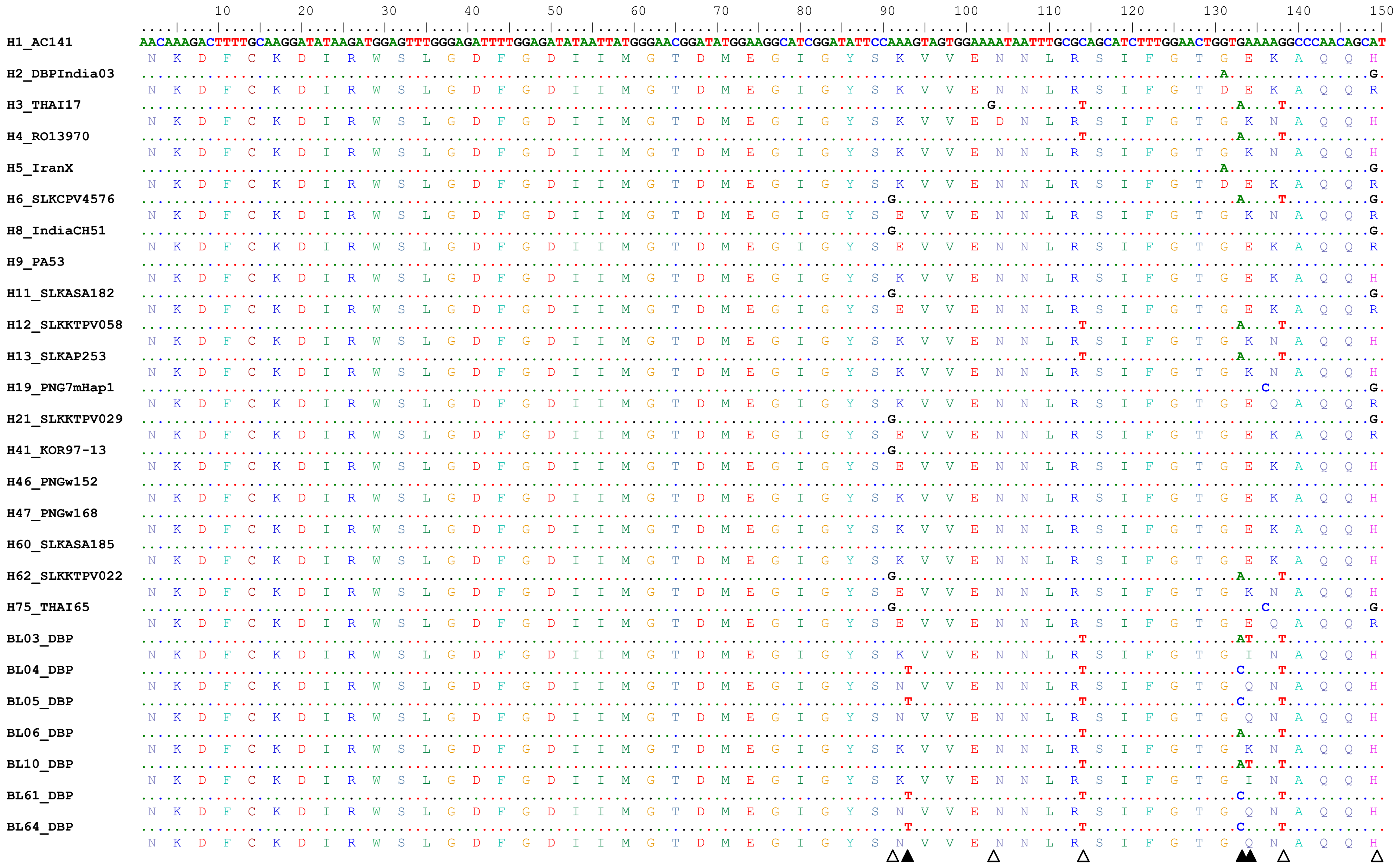
10 20 30 40 50 60 70 80 90 100 110 120 130 140 150
H1_AC141
AACAAAGACTTTTGCAAGGATATAAGATGGAGTTTGGGAGATTTTGGAGATATAATTATGGGAACGGATATGGAAGGCATCGGATATTCCAAAGTAGTGGAAAATAATTTGCGCAGCATCTTTGGAACTGGTGAAAAGGCCCAACAGCAT
N K D F C K D I R W S L G D F G D I I M G T D M E G I G Y S K V V E N N L R S I F G T G E K A Q Q H
H2_DBPIndia03
H3_THAI17
H4_RO13970
H5_IranX
H6_SLKCPV4576
H8_IndiaCH51
H9_PA53
H11_SLKASA182
H12_SLKKTPV058
H13_SLKAP253
H19_PNG7mHap1
H21_SLKKTPV029
H41_KOR97-13
H46_PNGw152
H47_PNGw168
H60_SLKASA185
H62_SLKKTPV022
H75_THAI65
BL03_DBP
BL04_DBP
BL05_DBP
BL06_DBP
BL10_DBP
BL61_DBP
BL64_DBP

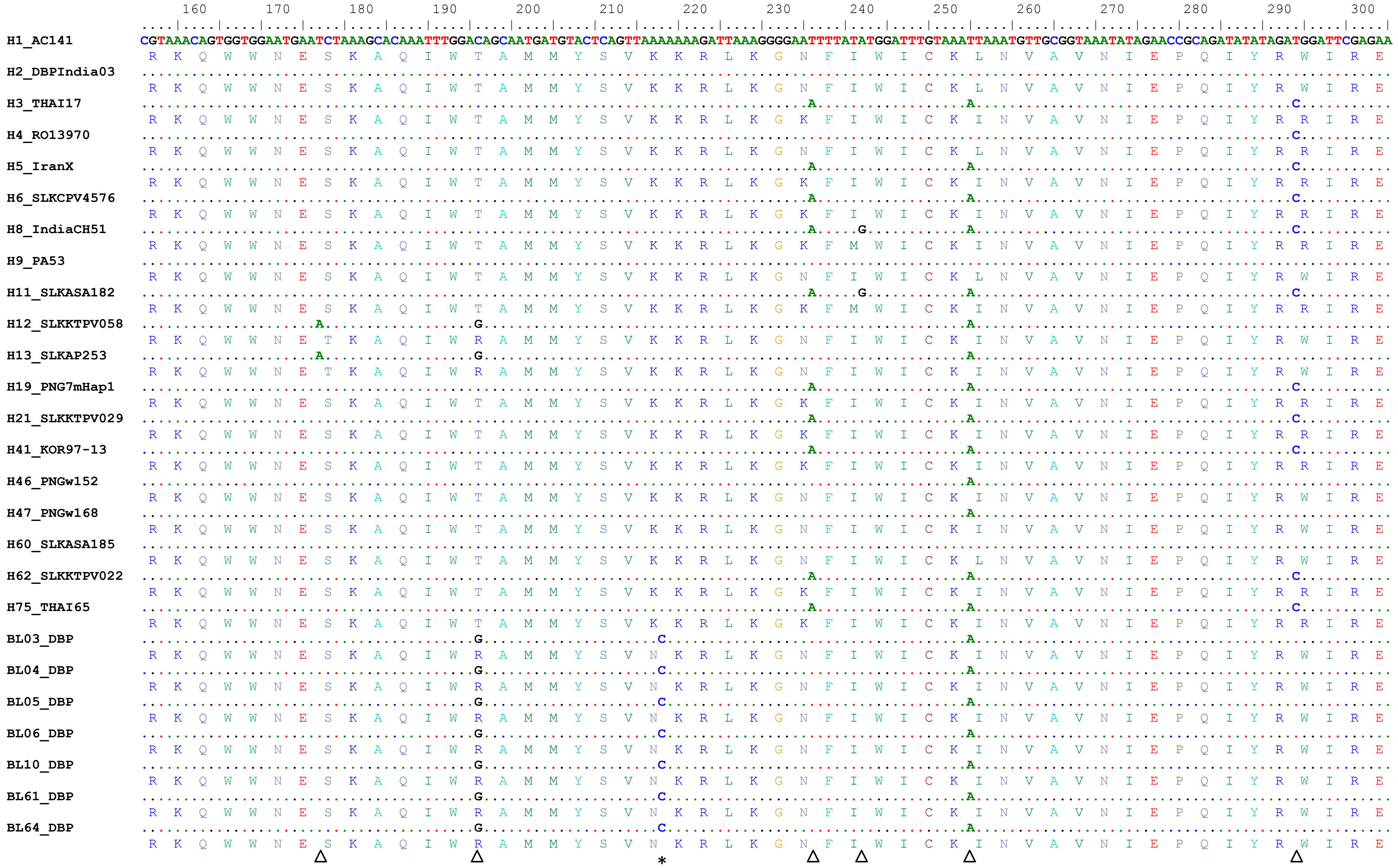
160 170 180 190 200 210 220 230 240 250 260 270 280 290 300
H1_AC141
CGTAAACAGTGGTGGAATGAATCTAAAGCACAAATTTGGACAGCAATGATGTACTCAGTTAAAAAAAGATTAAAGGGGAATTTTATATGGATTTGTAAATTAAATGTTGCGGTAAATATAGAACCGCAGATATATAGATGGATTCGAGAA
R K Q W W N E S K A Q I W T A M M Y S V K K R L K G N F I W I C K L N V A V N I E P Q I Y R W I R E
H2_DBPIndia03
H3_THAI17
H4_RO13970
H5_IranX
H6_SLKCPV4576
H8_IndiaCH51
H9_PA53
H11_SLKASA182
H12_SLKKTPV058
H13_SLKAP253
H19_PNG7mHap1
H21_SLKKTPV029
H41_KOR97-13
H46_PNGw152
H47_PNGw168
H60_SLKASA185
H62_SLKKTPV022
H75_THAI65
BL03_DBP
BL04_DBP
BL05_DBP
BL06_DBP
BL10_DBP
BL61_DBP
BL64_DBP

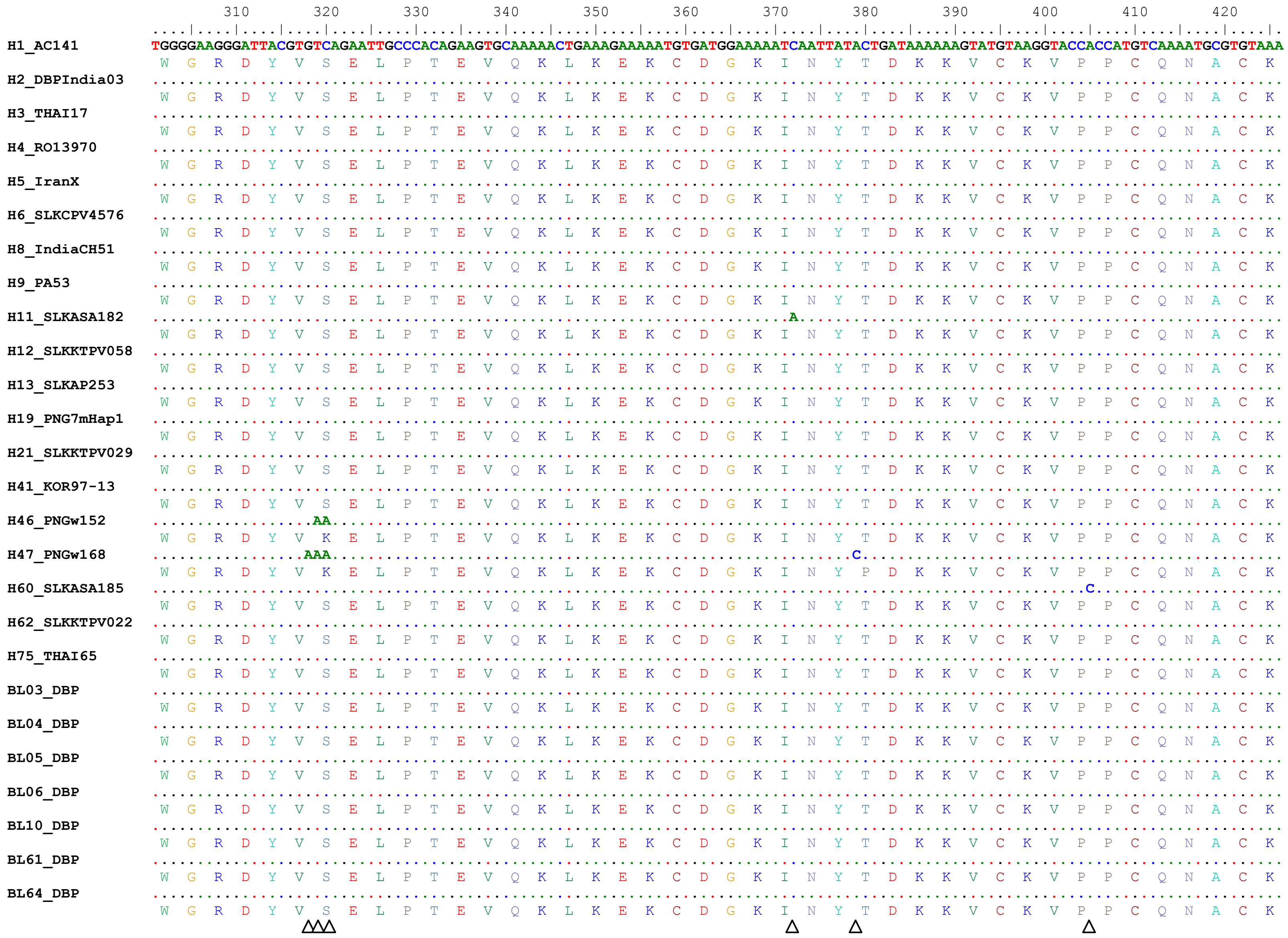
310 320 330 340 350 360 370 380 390 400 410 420
H1_AC141
TGGGGAAGGGATTACGTGTCAGAATTGCCCACAGAAGTGCAAAAACTGAAAGAAAAATGTGATGGAAAAATCAATTATACTGATAAAAAAGTATGTAAGGTACCACCATGTCAAAATGCGTGTAAA
W G R D Y V S E L P T E V Q K L K E K C D G K I N Y T D K K V C K V P P C Q N A C K
H2_DBPIndia03
H3_THAI17
H4_RO13970
H5_IranX
H6_SLKCPV4576
H8_IndiaCH51
H9_PA53
H11_SLKASA182
H12_SLKKTPV058
H13_SLKAP253
H19_PNG7mHap1
H21_SLKKTPV029
H41_KOR97-13
H46_PNGw152
H47_PNGw168
H60_SLKASA185
H62_SLKKTPV022
H75_THAI65
BL03_DBP
BL04_DBP
BL05_DBP
BL06_DBP
BL10_DBP
BL61_DBP
BL64_DBP
