# Supplementary material for: Plasmodium simium, a Plasmodium vivax-Related Malaria Parasite: Genetic Variability of Duffy Binding Protein II and the Duffy Antigen/Receptor for Chemokines
Source: PLoS One. 2015 Jun 24;10(6):e0131339. doi: 10.1371/journal.pone.0131339 (PMC4480967; doi:10.1371/journal.pone.0131339)
Supplement: S2 Fig — darc sequences of Alouatta g. clamitans were aligned with other primate darc sequences available in GenBank (nucleotides 1 to 768, from Homo sapiens darc sequence, accession number JN251915.1). The numbered bars above the sequences indicate transmembrane domains. The grey box shows the N-terminal minimum-binding domain (19–30 aa), and residues responsible for direct interaction with DBPII are underlined (20,21,22 and 24,25,26). Colored bars on the left side of the alignment represent the phylogenetic group families: red (Cercopithecidae), blue (Hominidae), green (Hylobatidae), purple (Pitheciidae), orange (Atelidae) and aqua (Cebidae). The arrow indicates the polymorphism Asp42Gly, which is responsible for the FY*A and FY*B alleles. (PDF) [file pone.0131339.s002.pdf]

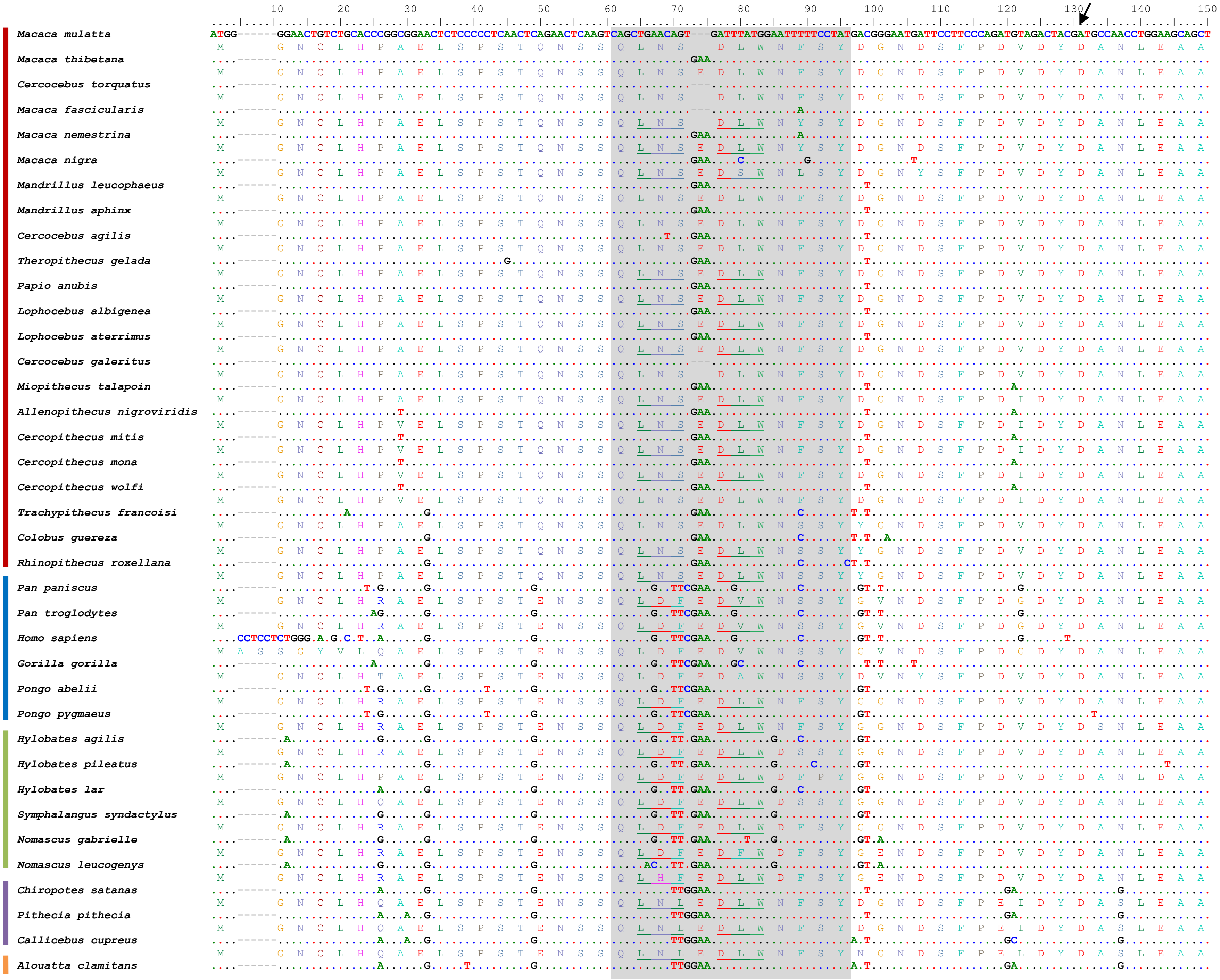

10 20 30 40 50 60 70 80 90 100 110 120 130 140 150
Macaca mulatta
Macaca thibetana
Cercocebus torquatus
Macaca fascicularis
Macaca nemestrina
Macaca nigra
Mandrillus leucophaeus
Mandrillus aphinx
Cercocebus agilis
Theropithecus gelada
Papio anubis
Lophocebus albigenea
Lophocebus aterrimus
Cercocebus galeritus
Miopithecus talapoin
Allenopithecus nigroviridis
Cercopithecus mitis
Cercopithecus mona
Cercopithecus wolfi
Trachypithecus francoisi
Colobus guereza
Rhinopithecus roxellana
Pan paniscus
Pan troglodytes
Homo sapiens
Gorilla gorilla
Pongo abelii
Pongo pygmaeus
Hylobates agilis
Hylobates pileatus
Hylobates lar
Symphalangus syndactylus
Nomascus gabrielle
Nomascus leucogenys
Chiropotes satanas
Pithecia pithecia
Callicebus cupreus
Alouatta clamitans

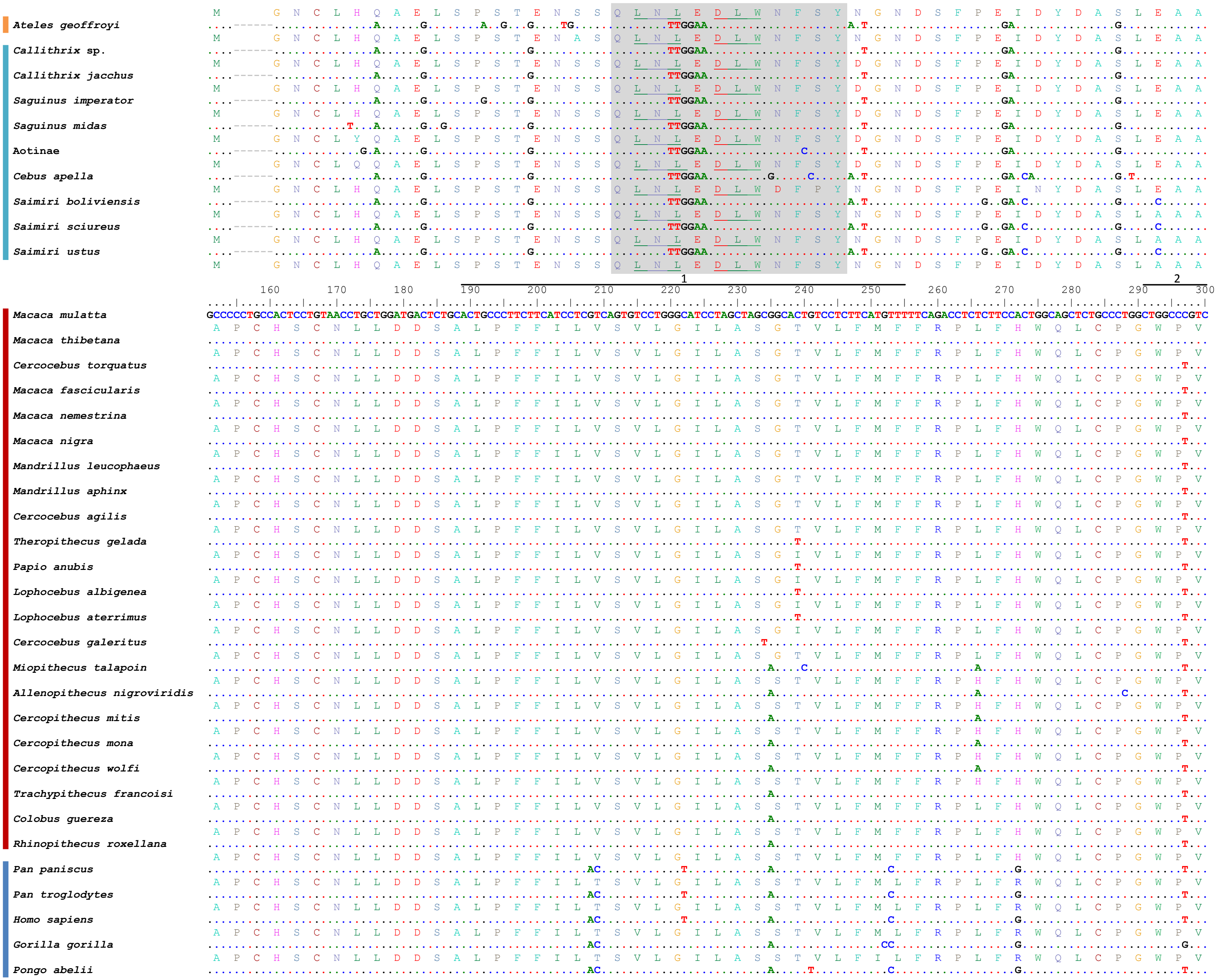
Ateles geoffroyi
Callithrix sp.
Callithrix jacchus
Saguinus imperator
Saguinus midas
Aotinae
Cebus apella
Saimiri boliviensis
Saimiri sciureus
Saimiri ustus
1
2
160 170 180 190 200 210 220 230 240 250 260 270 280 290 300
Macaca mulatta
Macaca thibetana
Cercocebus torquatus
Macaca fascicularis
Macaca nemestrina
Macaca nigra
Mandrillus leucophaeus
Mandrillus aphinx
Cercocebus agilis
Theropithecus gelada
Papio anubis
Lophocebus albigenea
Lophocebus aterrimus
Cercocebus galeritus
Miopithecus talapoin
Allenopithecus nigroviridis
Cercopithecus mitis
Cercopithecus mona
Cercopithecus wolfi
Trachypithecus francoisi
Colobus guereza
Rhinopithecus roxellana
Pan paniscus
Pan troglodytes
Homo sapiens
Gorilla gorilla
Pongo abelii

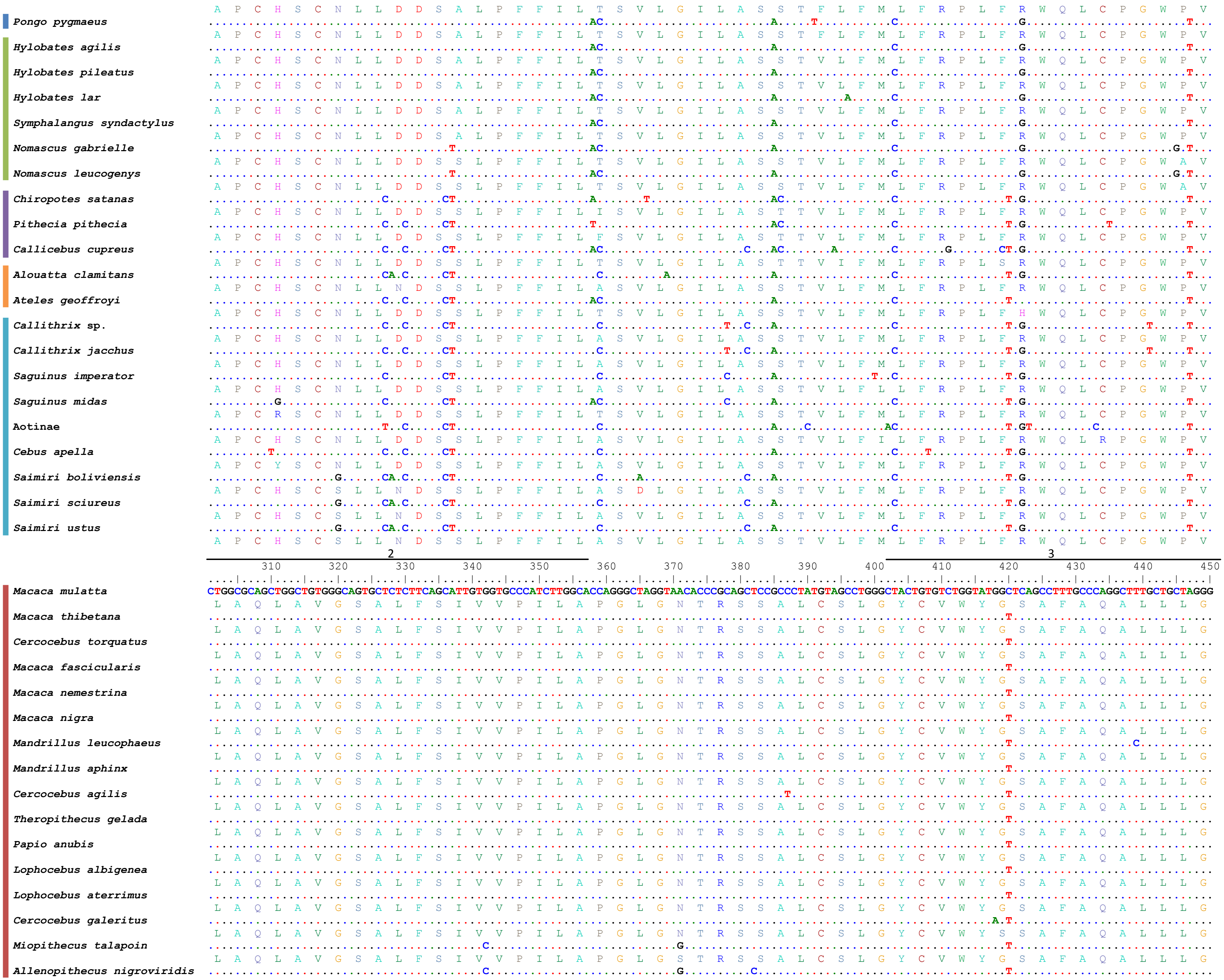

Pongo pygmaeus
Hylobates agilis
Hylobates pileatus
Hylobates lar
Symphalangus syndactylus
Nomascus gabrielle
Nomascus leucogenys
Chiropotes satanas
Pithecia pithecia
Callicebus cupreus
Alouatta clamitans
Ateles geoffroyi
Callithrix sp.
Callithrix jacchus
Saguinus imperator
Saguinus midas
Aotinae
Cebus apella
Saimiri boliviensis
Saimiri sciureus
Saimiri ustus
2
3
310 320 330 340 350 360 370 380 390 400 410 420 430 440 450
CTGGCGCAGCTGGCTGTGGGCAGTGCTCTCTTCAGCATTGTGGTGCCCATCTTGGCACCAGGGCTAGGTAACACCCGCAGCTCCGCCCTATGTAGCCTGGGCTACTGTGTCTGGTATGGCTCAGCCTTTGCCCAGGCTTTGCTGCTAGGG
Macaca mulatta
Macaca thibetana
Cercocebus torquatus
Macaca fascicularis
Macaca nemestrina
Macaca nigra
Mandrillus leucophaeus
Mandrillus aphinx
Cercocebus agilis
Theropithecus gelada
Papio anubis
Lophocebus albigenea
Lophocebus aterrimus
Cercocebus galeritus
Miopithecus talapoin
Allenopithecus nigroviridis

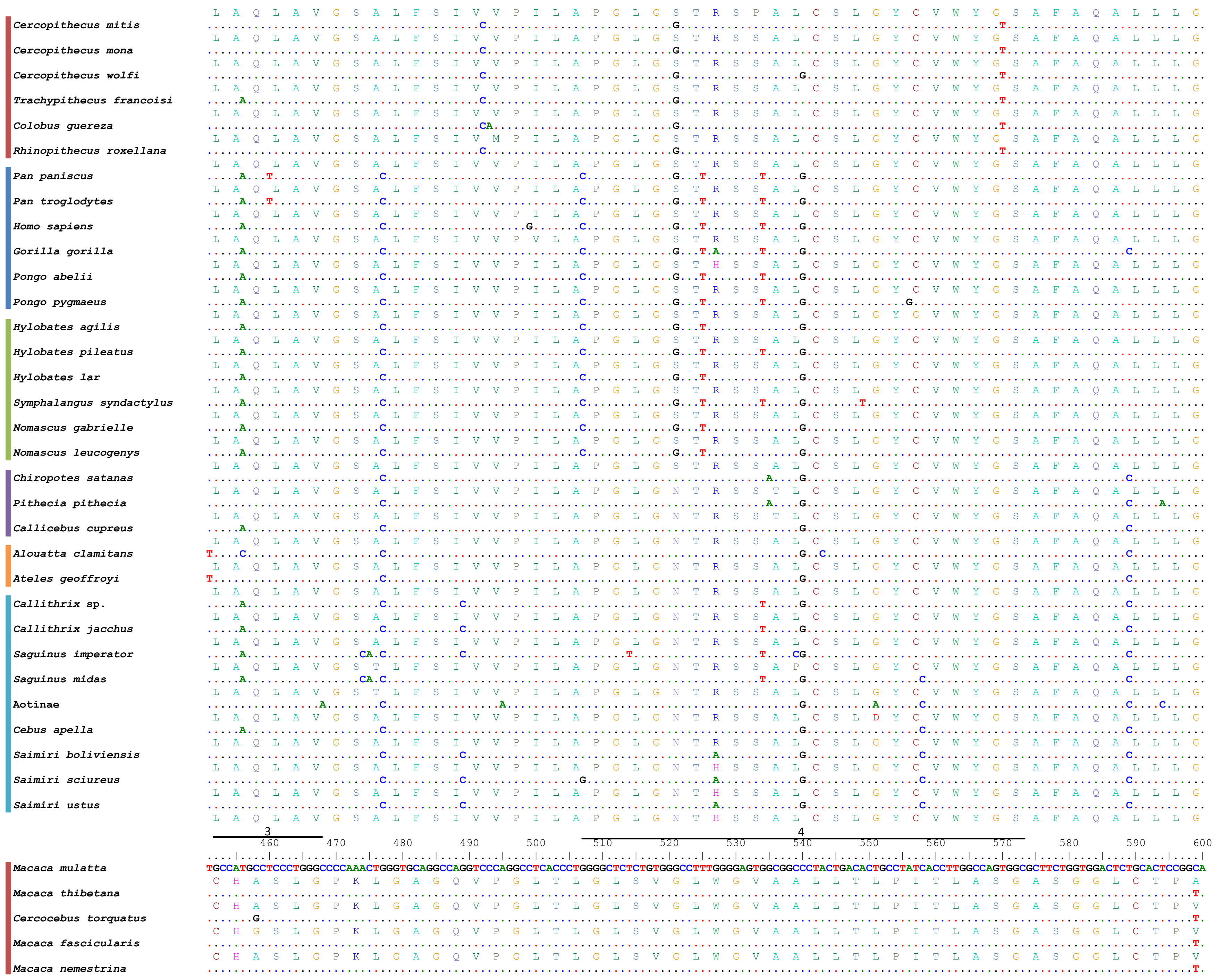

Cercopithecus mitis
Cercopithecus mona
Cercopithecus wolfi
Trachypithecus francoisi
Colobus guereza
Rhinopithecus roxellana
Pan paniscus
Pan troglodytes
Homo sapiens
Gorilla gorilla
Pongo abelii
Pongo pygmaeus
Hylobates agilis
Hylobates pileatus
Hylobates lar
Symphalangus syndactylus
Nomascus gabrielle
Nomascus leucogenys
Chiropotes satanas
Pithecia pithecia
Callicebus cupreus
Alouatta clamitans
Ateles geoffroyi
Callithrix sp.
Callithrix jacchus
Saguinus imperator
Saguinus midas
Aotinae
Cebus apella
Saimiri boliviensis
Saimiri sciureus
Saimiri ustus
3
4
460
470
480
490
500
510
520
530
540
550
560
570
580
590
600
Macaca mulatta
Macaca thibetana
Cercocebus torquatus
Macaca fascicularis
Macaca nemestrina

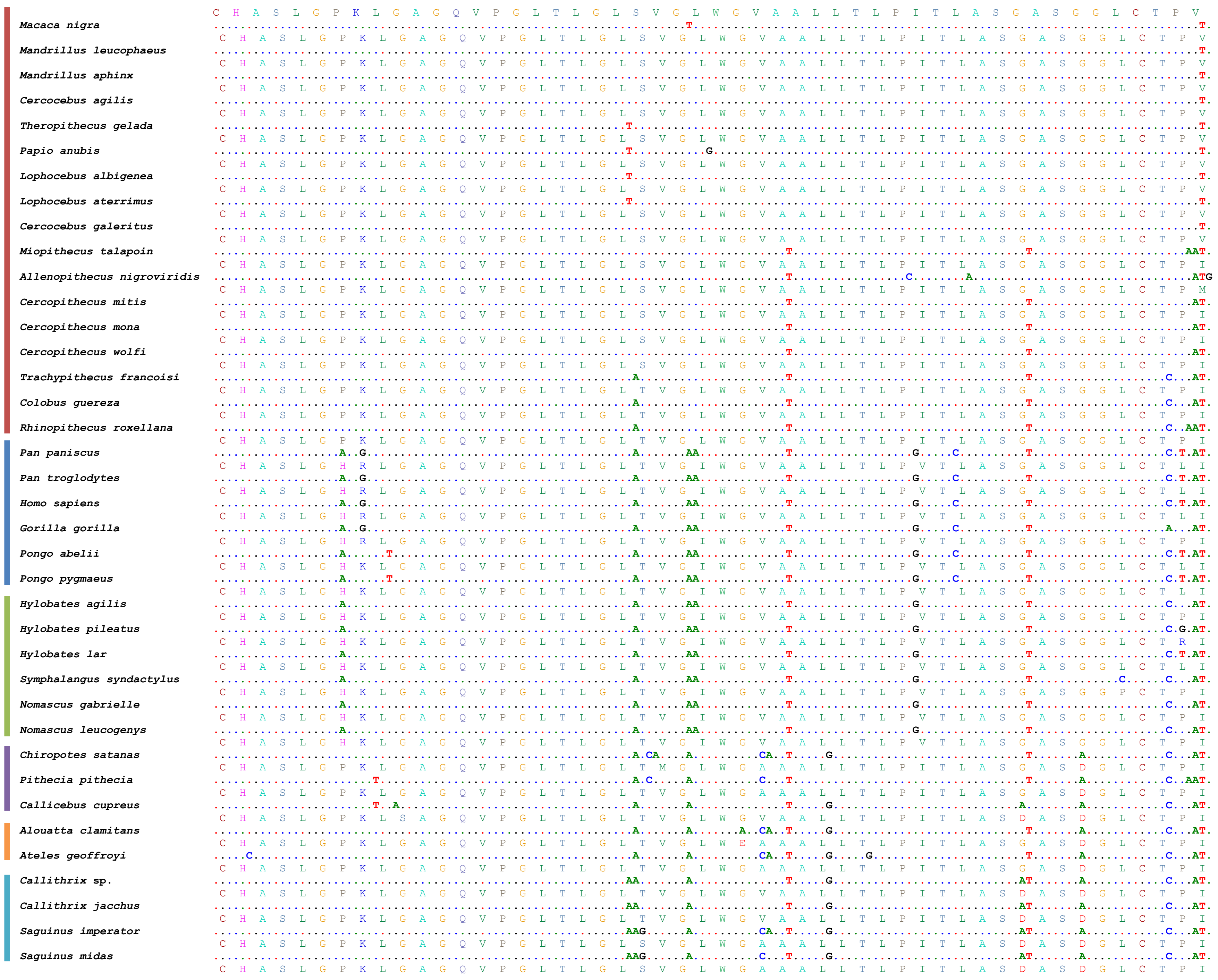

C H A S L G P K L G A G Q V P G L T L G L S V G L W G V A A L L T L P I T L A S G A S G G L C T P V
Macaca nigra
Mandrillus leucophaeus
Mandrillus aphinx
Cercocebus agilis
Theropithecus gelada
Papio anubis
Lophocebus albigenea
Lophocebus aterrimus
Cercocebus galeritus
Miopithecus talapoin
Allenopithecus nigroviridis
Cercopithecus mitis
Cercopithecus mona
Cercopithecus wolfi
Trachypithecus francoisi
Colobus guereza
Rhinopithecus roxellana
Pan paniscus
Pan troglodytes
Homo sapiens
Gorilla gorilla
Pongo abelii
Pongo pygmaeus
Hylobates agilis
Hylobates pileatus
Hylobates lar
Symphalangus syndactylus
Nomascus gabrielle
Nomascus leucogenys
Chiropotes satanas
Pithecia pithecia
Callicebus cupreus
Alouatta clamitans
Ateles geoffroyi
Callithrix sp.
Callithrix jacchus
Saguinus imperator
Saguinus midas

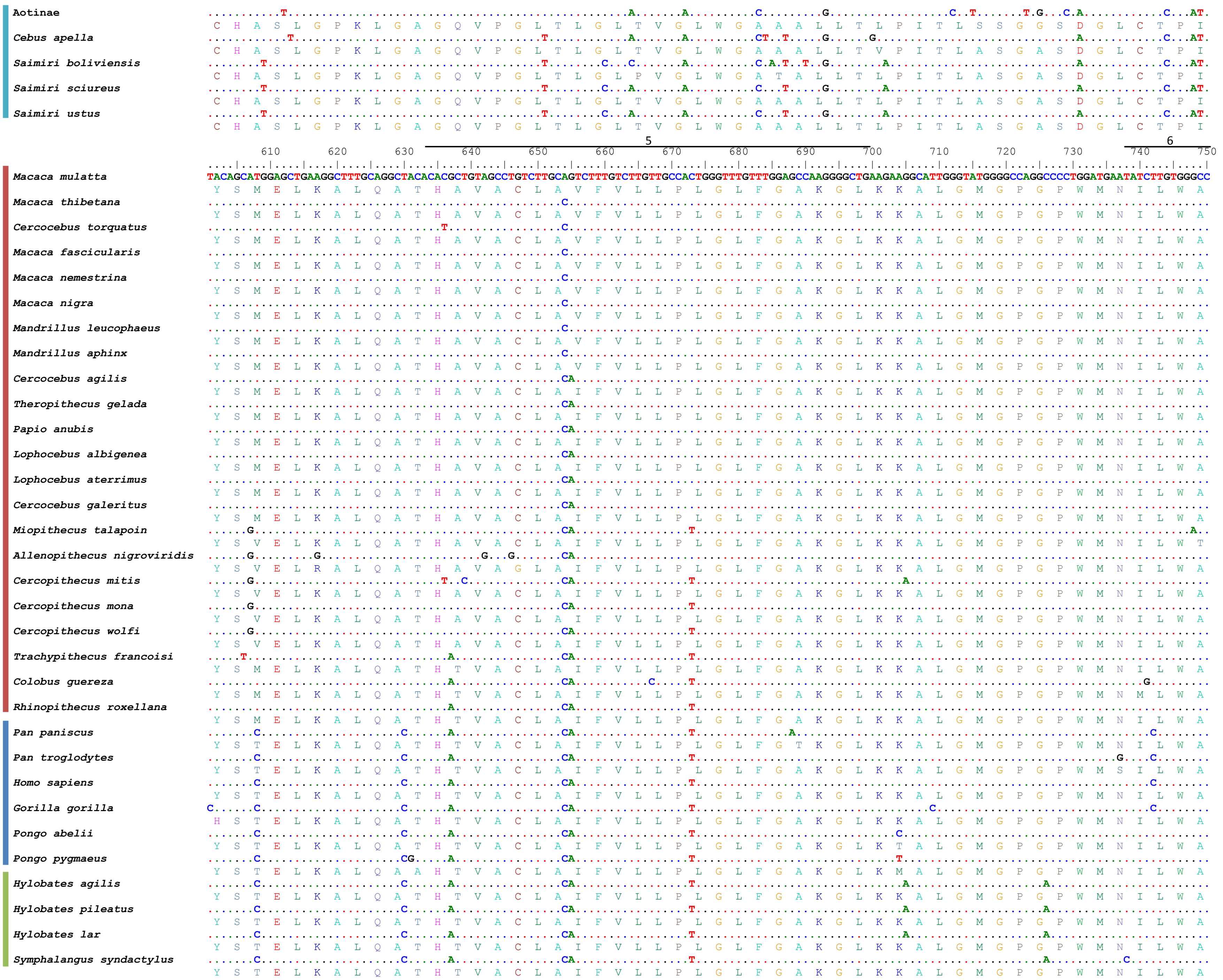
Aotinae
Cebus apella
Saimiri boliviensis
Saimiri sciureus
Saimiri ustus
5
6
Macaca mulatta
Macaca thibetana
Cercocebus torquatus
Macaca fascicularis
Macaca nemestrina
Macaca nigra
Mandrillus leucophaeus
Mandrillus aphinx
Cercocebus agilis
Theropithecus gelada
Papio anubis
Lophocebus albigenea
Lophocebus aterrimus
Cercocebus galeritus
Miopithecus talapoin
Allenopithecus nigroviridis
Cercopithecus mitis
Cercopithecus mona
Cercopithecus wolfi
Trachypithecus francoisi
Colobus guereza
Rhinopithecus roxellana
Pan paniscus
Pan troglodytes
Homo sapiens
Gorilla gorilla
Pongo abelii
Pongo pygmaeus
Hylobates agilis
Hylobates pileatus
Hylobates lar
Symphalangus syndactylus

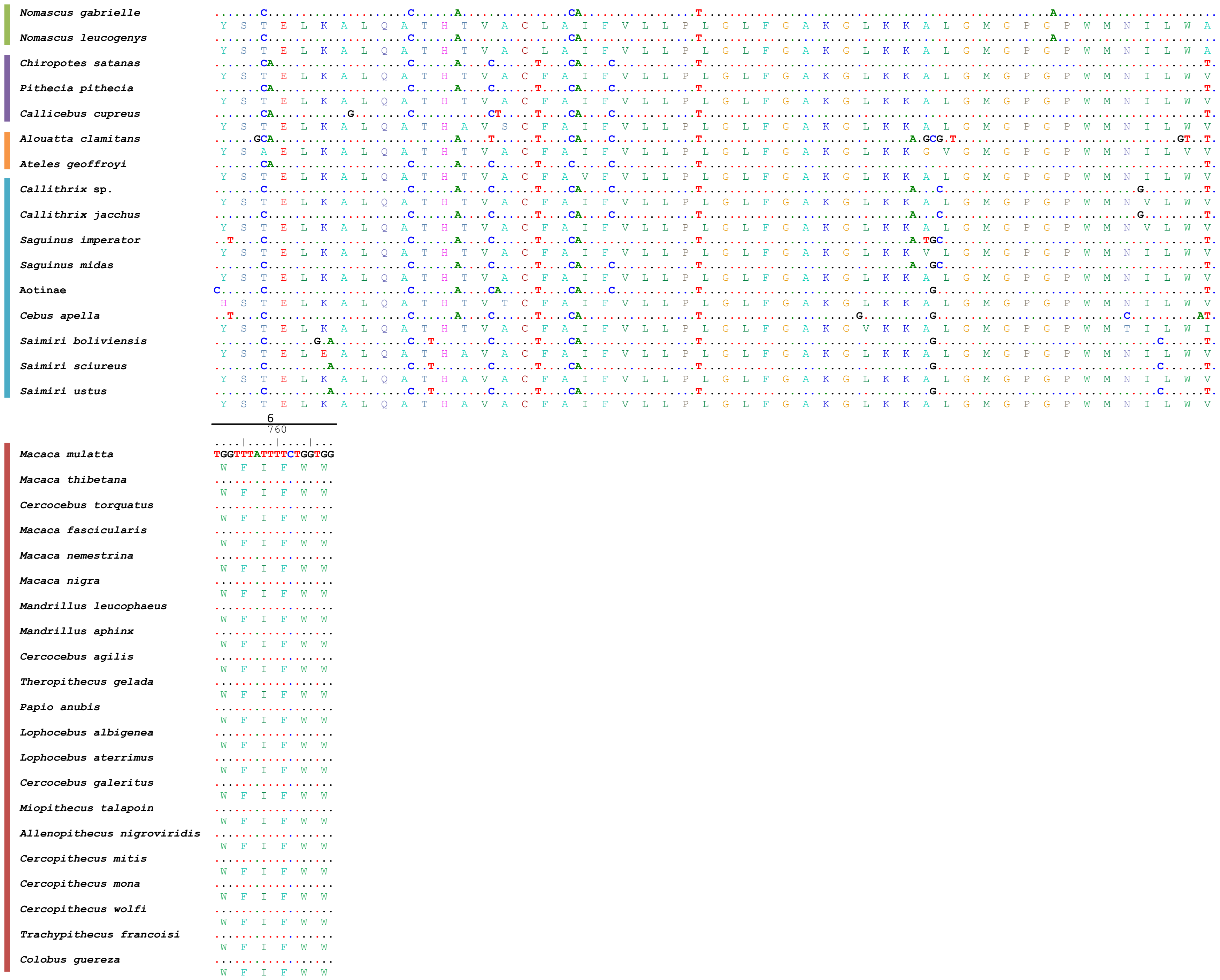
Nomascus gabrielle
Nomascus leucogenys
Chiropotes satanas
Pithecia pithecia
Callicebus cupreus
Alouatta clamitans
Ateles geoffroyi
Callithrix sp.
Callithrix jacchus
Saguinus imperator
Saguinus midas
Aotinae
Cebus apella
Saimiri boliviensis
Saimiri sciureus
Saimiri ustus
6
760
Macaca mulatta
TGGTTTATTTTCTGGTGG
W F I F W W
Macaca thibetana
Cercocebus torquatus
Macaca fascicularis
Macaca nemestrina
Macaca nigra
Mandrillus leucophaeus
Mandrillus aphinx
Cercocebus agilis
Theropithecus gelada
Papio anubis
Lophocebus albigenea
Lophocebus aterrimus
Cercocebus galeritus
Miopithecus talapoin
Allenopithecus nigroviridis
Cercopithecus mitis
Cercopithecus mona
Cercopithecus wolfi
Trachypithecus francoisi
Colobus guereza

Rhinopithecus roxellana	....	W F I F W W
Pan paniscus	....	W F I F W W
Pan troglodytes	....	W F I F W W
Homo sapiens	....	W F I F W W
Gorilla gorilla	....	W F I F W W
Pongo abelii	....	W F I F W W
Pongo pygmaeus	....	W F I F W W
Hylobates agilis	....	W F I F W W
Hylobates pileatus	....	W F I F W W
Hylobates lar	....	W F I F W W
Symphalangus syndactylus	....	W F I F W W
Nomascus gabrielle	....	W F I F W W
Nomascus leucogenys	....	W F I F W W
Chiropotes satanas	....	W F I F W W
Pithecia pithecia	...............G..	W F I F W G
Callicebus cupreus	....	W F I F W W
Alouatta clamitans	....	W F I F W W
Ateles geoffroyi	....	W F I F W W
Callithrix sp.	....	W F I F W W
Callithrix jacchus	....	W F I F W W
Saguinus imperator	....	W F I F W W
Saguinus midas	....	W F I F W W
**Aotinae**	....	W F I F W W
Cebus apella	....	W F I F W W
Saimiri boliviensis	....	W F I F W W
Saimiri sciureus	....	W F I F W W
Saimiri ustus	....	W F I F W W
