# Supplementary figures and images for: Plasmodium simium, a Plasmodium vivax-Related Malaria Parasite: Genetic Variability of Duffy Binding Protein II and the Duffy Antigen/Receptor for Chemokines
Source: PLoS One. 2015 Jun 24;10(6):e0131339. doi: 10.1371/journal.pone.0131339 (PMC4480967; doi:10.1371/journal.pone.0131339)

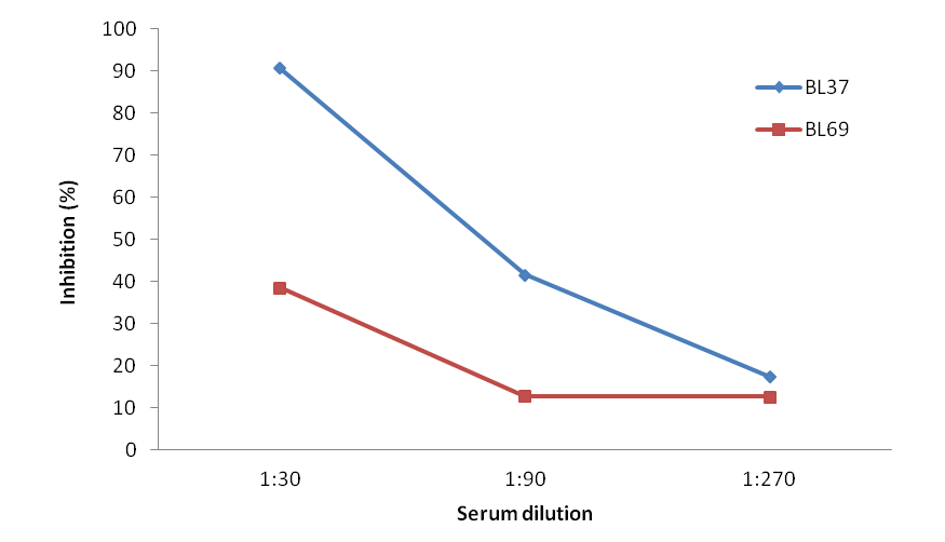

Supplement: S3 Fig — The sera from two monkeys (BL37 and BL69), which showed the highest levels of blockade, were used in dilutions of 1:30, 1:90 and 1:270 in the inhibition assays of human DARC and PvDBPII. Inhibition was calculated based on the reduction in the rosette numbers observed in the presence of monkey serum compared to the rosette numbers in the absence of serum. (TIF) [file pone.0131339.s003.tif]
